# Gene- or region-based association study via kernel principal component analysis

**DOI:** 10.1186/1471-2156-12-75

**Published:** 2011-08-26

**Authors:** Qingsong Gao, Yungang He, Zhongshang Yuan, Jinghua Zhao, Bingbing Zhang, Fuzhong Xue

**Affiliations:** 1 Department of Epidemiology and Health Statistics, School of Public Health, Shandong University, Jinan 250012, China; 2 CAS-MPG Partner Institute for Computational Biology, Shanghai Institutes for Biological Sciences, Chinese Academy of Sciences, Shanghai 200031, China; 3 Key Laboratory of Computational Biology, CAS-MPG Partner Institute for Computational Biology, Chinese Academy of Sciences, Shanghai 200031, China; 4 MRC Epidemiology Unit, Institute of Metabolic Science, Addenbrooke's Hospital, Cambridge, UK

## Abstract

**Background:**

In genetic association study, especially in GWAS, gene- or region-based methods have been more popular to detect the association between multiple SNPs and diseases (or traits). Kernel principal component analysis combined with logistic regression test (KPCA-LRT) has been successfully used in classifying gene expression data. Nevertheless, the purpose of association study is to detect the correlation between genetic variations and disease rather than to classify the sample, and the genomic data is categorical rather than numerical. Recently, although the kernel-based logistic regression model in association study has been proposed by projecting the nonlinear original SNPs data into a linear feature space, it is still impacted by multicolinearity between the projections, which may lead to loss of power. We, therefore, proposed a KPCA-LRT model to avoid the multicolinearity.

**Results:**

Simulation results showed that KPCA-LRT was always more powerful than principal component analysis combined with logistic regression test (PCA-LRT) at different sample sizes, different significant levels and different relative risks, especially at the genewide level (1E-5) and lower relative risks (RR = 1.2, 1.3). Application to the four gene regions of rheumatoid arthritis (RA) data from Genetic Analysis Workshop16 (GAW16) indicated that KPCA-LRT had better performance than single-locus test and PCA-LRT.

**Conclusions:**

KPCA-LRT is a valid and powerful gene- or region-based method for the analysis of GWAS data set, especially under lower relative risks and lower significant levels.

## Background

It is commonly believed that genetic factors play an important role in the etiology of common diseases and traits. With rapid improvements in high-throughout genotyping techniques and the growing number of available markers, genome-wide association studies (GWAS) have been promising approaches for identifying common genetic variants. The first successful wave of GWAS has reproducibly identified hundreds of associations of common genetic variants with more than 100 diseases and traits, including age-related macular degenerative diseases [1], Parkinson's disease [2] and type 2 diabetes [3,4]. Recently GWAS meta-analysis, which combines the evidence for association from individual studies with appropriate weights, is becoming an increasingly important method to identify new loci of complex disease and traits [5-7]. Although this has improved our understanding of the genetic basis of these complex diseases and traits, and has provided valuable clues to their allelic architecture, there are still many analytic and interpretation challenges in GWAS [8-11]. For both GWAS and GWAS meta-analysis, it is customary to run single-locus association tests in the whole genome to identify causal or associated single nucleotide polymorphisms (SNPs) with strong marginal effects on disease or traits. However, such a SNP-by-SNP analysis leads to computational burden and the well-known multiplicity problem, with a highly inflated risk of type I error and decreased ability to detect modest effects. One way to deal with these and related challenges is to consider higher units for the analysis such as genes or regions. Several studies have shown that treating gene or region instead of SNP as the unit of association may alleviate the problems of intensive computation and multiple testing [8,10], lead to more stable results and higher interpretability [12,13], be regarded as good standards for subsequent replication studies [14] and suit for network (or pathway) approaches to interpret the finds from GWAS [15].

However, given the SNPs allocated into genes or regions, the issue of how to evaluate genetic association for each candidate gene or genome region remains. To examine whether multiple SNPs in the candidate gene or region are associated with disease or trait, several multi-marker analysis methods have been developed, including haplotype-based methods [16,17], Hotelling's *T*^2 ^test [18,19], principal component analysis (PCA)-based methods [20-23], and P-value combination methods [11,24,25]. Especially, the PCA-based methods have been shown to be as or more powerful than standard joint SNP or haplotype-based tests [23]. PCA can capture linkage disequilibrium information within a candidate gene/region, but is less computationally demanding compared to haplotype-based analysis. It also avoids multicolinearity between SNPs, for the principal components (PCs) are orthogonal.

However, one cannot assert that linear PCA will always detect all structure in a given genomic data set. If the genomic data contains nonlinear structure, PCA will not be able to detect it [26]. Furthermore, it is well known that PCA can not accurately represent non-Gaussian distributions. Up to now, many researchers have introduced appropriate nonlinear process into PCA and developed nonlinear PCA algorithms [27-31]. Among these modified PCA methods, the kernel PCA (KPCA) is the most well known and widely adopted [27-30], which has several advantages than other methods: (1) it does not require nonlinear optimization, but just the solution of an eigenvalue problem; (2) it provides a better understanding of what kind of nonlinear features are extracted: they are principal components in a feature space which is fixed a priori by choosing a kernel function; (3) it comprises a fairly general class of nonlinearities by the possibility to use different kernels.

KPCA has been studied intensively in the last several years in the field of machine learning, face recognition and data classification, and has been claimed success in many applications [27-30]. Especially, for classifying tumour samples, Liu et al proposed to combine KPCA with logistic regression test (KPCA-LRT) by gene expression data [30]. Nevertheless, the purpose of association study is to detect the correlation between genetic variations and disease rather than to classify the sample, and the genomic data is categorical rather than numerical. Recently, Wu et al proposed a kernel-based logistic regression model to detect the association between multiple SNPs and disease by projecting the nonlinear original SNPs data into a linear feature space [32]. However, the logistic model is still impacted by multicolinearity between the projections, which may lead to loss of power. We, therefore, propose a KPCA-LRT model to avoid the multicolinearity. The algorithm conducts KPCA first to account for the nonlinear relationship between SNPs in a candidate region, and then apply LRT to test the association between kernel principal components (KPCs) scores and diseases. Simulations and real data application are conducted to evaluate its performance in association study.

## Methods

### PCA

As a traditional multivariable statistical technique, PCA has been widely applied in genetic analysis, both for reduction of redundant information and interpretation of multiple SNPs. The basic idea of PCA is to efficiently represent the data by decomposing a data space into a linear combination of a small collection of bases consisting of orthogonal axes that maximally decorrelate the data. Assuming that *M *SNPs in a candidate gene or specific genome region of interests have coded values {*x*_*i *_∈ *R*^*M *^| *i *= 1,2,...,*N*}, where *N *represents sample size giving a genetic model (assuming additive model here). PCA diagonalizes the covariance matrix of the centered observations *x*_*i*_, $\overset{N}{\underset{i=1}{\sum }}x_{i}=0$, defined as

(1) $$C=\frac{1}{N}\overset{N}{\underset{i=1}{\sum }}x_{i}x_{i}^{T}$$

To do this, one has to solve the following eigenvalue problem:

(2) Cv=λv

where *ν *are the eigenvectors of *C*, and λ are the corresponding eigenvalues. As $Cv=\frac{1}{N}\overset{N}{\underset{i=1}{\sum }}\left(x_{i}\cdot v\right)x_{i}$, all solutions *ν *must lie in the span of {*x*_*i *_∈ *R*^*M *^| *i *= 1,2,...,*N*}, hence (2) is equivalent to

$$\lambda \left(x_{i}\cdot v\right)=x_{i}\cdot Cv\;\text{for}\;\text{all}\;i=1,\;2,\;...,\;N,$$

where the dot product of two vectors *a *= (*a*_1_, *a*_2_, ..., *a*_*N*_) and *b *= (*b*_1_, *b*_2_, ..., *b*_*N*_) is defined as $a\cdot b=\overset{N}{\underset{i=1}{\sum }}a_{i}b_{i}=a_{1}b_{1}+a_{2}b_{2}+\cdots +a_{N}b_{N}$.

### KPCA

Given the observations, we first map the data nonlinearly into a feature space *F *by

$$\begin{array}{c} \Phi :R^{M}\to F\\ \;\;x\to X. \end{array}$$

Again, we make the assumption that our data mapped into feature space, Φ(*x*_1_),...,Φ(*x*_*N*_), is centered, i.e. $\overset{N}{\underset{i=1}{\sum }}\Phi \left(x_{i}\right)=0$. To do PCA for the covariance matrix

$$\bar{C}=\frac{1}{N}\overset{N}{\underset{i=1}{\sum }}\Phi \left(x_{i}\right)\Phi {\left(x_{i}\right)}^{T}$$

we have to find eigenvalues *λ *≥ 0 and eigenvectors ν ∈ *F*\{0} satisfying

(3) $$\bar{C}v=\lambda v.$$

By the same argument as above, the solutions *ν *lie in the span of Φ(*x*_1_),...,Φ(*x*_*N*_). This implies that we may consider the equivalent equation

(4) $$\lambda \left(\Phi \left(x_{i}\right)\cdot v\right)=\left(\Phi \left(x_{i}\right)\cdot \bar{C}v\right)\;\text{for}\;\text{all}\;i=1,\;2,\;...,\;N$$

and that there exist coefficients *a*_*i *_(*i *= 1,...,*N*) such that

(5) $$v=\overset{N}{\underset{i=1}{\sum }}\alpha_{i}\Phi \left(x_{i}\right).$$

Substituting (3) and (5) into (4), we arrive at

(6) $$K^{2}\alpha =N\lambda K\alpha$$

where *α *denotes the column vector with entries *α*_1_, ..., *α*_*N*_, and *K *is a symmetric *N *× *N *matrix defined by

(7) $$K_{ij}:=\left(\Phi \left(x_{i}\right)\cdot \Phi \left(x_{j}\right)\right),$$

It has a set of eigenvectors which spans the whole space, thus

(8) Kα=Nλα

gives all solutions *α *of equation (6).

Assume *λ*_1 _≤ *λ*_2 _≤ ... ≤ *λ*_*N *_represent the eigenvalues for the matrix *K *with *α*^1^*, α*^2^*, ..., α*^*N *^being the corresponding complete set of eigenvectors. *λ*_*p *_is the first nonzero eigenvalue. We do the normalization for the solutions *α*^*p*^, ..., *α*^*N *^by requiring that the corresponding vectors in *F *be normalized, i.e. *ν*^*k *^· *ν*^*k *^= 1 for all *k *= *p, p *+ 1, ..., *N*. Based on (5), (6) and (8), this translates into

(9) $$\begin{array}{c} 1=\sum \overset{k}{\underset{i}{\alpha }}\alpha_{j}^{k}\left(\Phi \left(x_{i}\right)\cdot \Phi \left(x_{j}\right)\right)\\ \;\;=\left(\alpha^{k}\cdot K\alpha^{k}\right)\\ \;\;=\lambda_{k}\left(\alpha^{k}\cdot \alpha^{k}\right) \end{array}$$

We need to compute projections on the eigenvectors ν^*k *^in *F *to do principal component extraction. Suppose *x *is the SNP set within previously defined gene or genome region of an individual, with an image Φ(*x*) in *F*, then

(10) $$\left(v^{k}\cdot \Phi \left(x\right)\right)=\overset{N}{\underset{i=1}{\sum }}\alpha_{i}^{k}\left(\Phi \left(x_{i}\right)\cdot \Phi \left(x\right)\right)$$

are its nonlinear principal components corresponding to Φ.

Note that neither (7) nor (10) requires Φ(*x*_*i*_) in explicit form - they are only needed in dot products. We, therefore, are able to use kernel functions for computing these dot products without actually performing the map Φ: for some choices of a kernel *k*(*x*_*i*_, *x*_*j*_), by methods of functional analysis, it can be shown that there exists a map Φ into some dot product space *F *(possibly of infinite dimension) such that *k*(*x*_*i*_, *x*_*j*_) can compute the dot product in *F*. This property is often called "kernel trick" in the literature.

Theoretically, a proper function can be created for each data set based on the Mercer's theorem of functional analysis [29]. The most common kernel functions include linear kernel, polynomial kernel, radial basis function (RBF) kernel, sigmoid kernel [30], IBS kernel and weighted IBS kernel [32]. In particular, KPCA with linear kernel is the same as standard linear PCA. It is worth noting that in general, the above kernel functions show similar performance if appropriate parameters are chosen. In present work, we chose the RBF kernel owing to its flexibility in choosing the associated parameter [33].

There are two widely used approaches for the selection of parameters for a certain kernel function. The first method chooses a series of candidate values for the concerned kernel parameter empirically, performs the learning algorithm using each candidate value, and finally assigns the value based on the best performance to the kernel parameter. As is well-known to us, the second one is the cross-validation. However, both approaches are time-consuming and with high computation burden [34]. For RBF kernel applied in present study, there is a popular way of choosing the bandwidth parameter σ, which is to set it to the median of all pairwise Euclidean distances ||*x*_*i *_- *x*_*j*_|| in the set {*x*_*k *_∈ *R*^*M *^| *k *= 1, 2, ..., *N*} for all 1 ≤ *i *<*j *≤ *N *[35-37].

### Models

To test the associations between multiple SNPs and disease, the PCA-LRT and KPCA-LRT models are defined as follows:

(11) $$\begin{array}{c} \;Logit\left[\mathrm{Pr}\left(D=1|PC_{1},\;PC_{2},\;\ldots ,\;PC_{L}\right)\right]\\ =\beta_{0}+\beta_{1}PC_{1}+\cdots +\beta_{L}PC_{L} \end{array}$$

(12) $$\begin{array}{c} \;Logit\left[\mathrm{Pr}\left(D=1|KPC_{1},\;KPC_{2},\;\ldots ,\;KPC_{L}\right)\right]\\ =\beta_{0}+\beta_{1}KPC_{1}+\cdots +\beta_{L}KPC_{L} \end{array}$$

where *PCs *and *KPCs *are the first *L*^*th *^linear and nonlinear (kernel) principal component scores of the SNPs, respectively. The value of *L *can be chosen such that the cumulative contributing proportion of the total variability explained by the first *L *PCs (*λ*_1 _*+ λ*_2 _*+ ···+ λ*_*L*_)/(*λ*_1 _*+ λ*_2 _*+ ··· + λ*_*M*_) exceeds some threshold. For comparison, we set the same threshold of 80% in both PCA-LRT and KPCA-LRT as Gauderman et al [34].

### Data simulation

**Figure 1:**
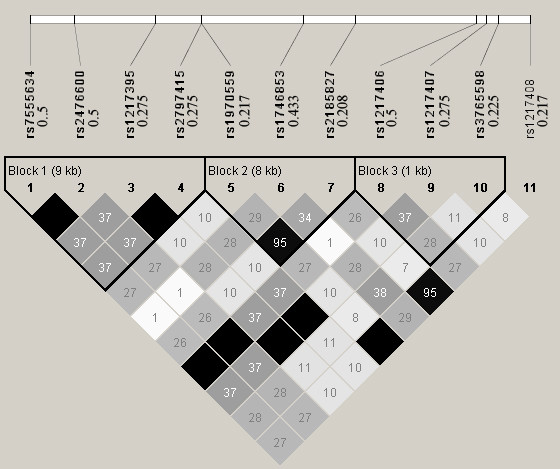
**Pairwise R**^**2 **^**among the 11 SNPs in the selected region**. The 11 SNPs are: rs7555634, rs2476600, rs1217395, rs2797415, rs1970559, rs1746853, rs2185827, rs1217406, rs1217407, rs3765598, rs1217408. The triangles mark the three haplotype blocks within this region. The value in each diamond is the R^2 ^value and the shading indicates the level of LD between a given pair of SNPs. The values to the right of the 11 dbSNP IDs (rs# IDs) are the corresponding minor allele frequencies.

To assess the performance of KPCA-LRT and compare it with PCA-LRT, we apply a statistical simulation based on HapMap data under the null hypothesis (*H*_0_) and alternative hypothesis (*H*_1_). The corresponding steps for the simulation are as follows:

*Step 1*. Download the phased haplotype data of a genome region from the HapMap web site (http://snp.cshl.org): we select the Protein tyrosine phosphatase, non-receptor type 22 (PTPN22) gene region to generate the simulating genotype data of *CEU *population using HapMap Phase 1& 2 full dataset. This region is located at Chr 1: 114168639..114197803, including 11 SNPs. Figure 1 shows their pair-wise R^2 ^structure and minor allele frequencies (MAF).

*Step 2*. Based on the HapMap phased haplotype data, we generate large samples with 100 000 cases and 100 000 controls as *CEU *populations using the software HAPGEN [38]. To investigate the performance of the two methods on different causal SNPs with different MAF and different LD patterns, each of the 11 SNPs was defined as the causal variant. We remove the causal SNP in the simulation to assess the indirect association with disease via correlated markers,. Under *H*_0_, we set the relative risk per allele as 1.0 to assess the type I error. Under *H*_1_, different levels of relative risks are set (1.1, 1.2, 1.3, 1.4 and 1.5 per allele) to assess the power. The SNPs in this region are coded according to the additive genetic model.

*Step 3*. From the remained SNPs, we sample the simulation data and perform the PCA-LRT and KPCA-LRT under different sample sizes *N *(*N*/2 cases and *N*/2 controls, *N *= 1000, 2000, ..., 12000) using the R packages *kernlab *(http://cran.r-project.org/web/packages/kernlab/index.html) and Design (http://cran.r-project.org/web/packages/Design/index.html). Under *H*_0_, we repeat 10 000 simulations at two significant levels (0.05 and 0.01). Under *H*_1_, for each model with a given relative risk, we repeat 10 000 simulations at four significant levels (0.05, 0.01, 1E-5 and 1E-7).

### Application

The proposed method is applied to rheumatoid arthritis (RA) data from GAW16 Problem 1. The data consists of 2062 Illumina 550 k SNP chips from 868 RA patients and 1194 normal controls collected by the North American Rheumatoid Arthritis Consortium (NARAC) [39]. At present study, only 1493 females (641 cases and 852 controls) are analyzed to avoid potential bias with the fact that rheumatoid arthritis is two to three times more common in women than in men [40].

To illustrate the performance of PCA-LRT and KPCA-LRT, we mainly focus on four special regions in chromosome 1, within the genes PTPN22, ANKRD35, DUSP23, RNF186 involved, respectively. The reasons are as follows: 1) Both the PTPN22 gene (R620W, rs2476601) and ANKRD35 gene have been reported to be associated with RA [41-43]; 2) DUSP23 can activate mitogen-activated protein kinase kinase [43], which may regulate a pathway in rheumatoid arthritis [44,45]; 3) RNF186 involves a ulcerative colitis-risk loci (rs3806308) [44], and RA may be associated with ulcerative colitis [45].

## Results

### Data simulation

#### Type I error

Simulation results under *H*_0 _are shown in Table 1, which indicates that the type I error rates of both PCA-LRT and KPCA-LRT are very close to given nominal values (*α *= 0.01, *α *= 0.05) under different sample sizes. This suggests that both the models perform well under null hypothesis.

**Table 1:** Type I error of PCA-LRT and KPCA-LRT

Sample size	PCA-LRT		KPCA-LRT	
	
	α = 0.05	α = 0.01	α = 0.05	α = 0.01
1000	0.052	0.011	0.049	0.012
2000	0.051	0.010	0.054	0.011
3000	0.056	0.011	0.052	0.012
4000	0.048	0.014	0.051	0.011
5000	0.053	0.012	0.050	0.010
6000	0.048	0.011	0.050	0.009
7000	0.051	0.009	0.052	0.011
8000	0.051	0.012	0.050	0.012
9000	0.051	0.008	0.051	0.012
10000	0.051	0.011	0.052	0.012
11000	0.050	0.011	0.051	0.011
12000	0.051	0.009	0.051	0.009

#### Power

**Figure 2:**
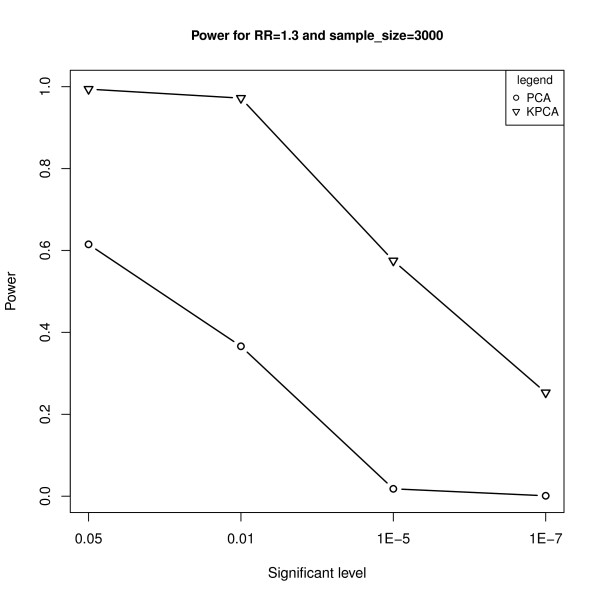
**The powers of PCA-LRT and KPCA-LRT under different significant levels at the given relative risk of 1.3 and sample size of 3000**. The horizontal axis denotes the significant levels and the vertical axis denotes the powers of PCA-LRT and KPCA-LRT.

When defining the 6^*th *^SNP (rs1746853) as the causal variant, Figure 2 shows the powers of the two models under different significant levels at the given relative risk of 1.3 and sample size of 3000. It is clear that KPCA-LRT is always much more powerful than PCA-LRT, especially at the significant level of 1E-5 (the suggested genewide level in Neale and Sham [14]). In the following, only the results at the significant level of 1E-5 are presented.

**Figure 3:**
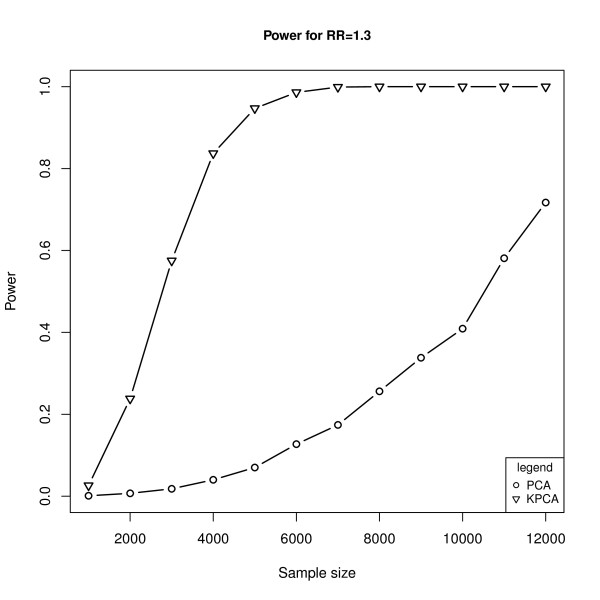
**The powers of PCA-LRT and KPCA-LRT under different sample sizes at the given relative risk of 1.3**. The horizontal axis denotes the sample sizes and the vertical axis denotes the powers of PCA-LRT and KPCA-LRT.

**Figure 4:**
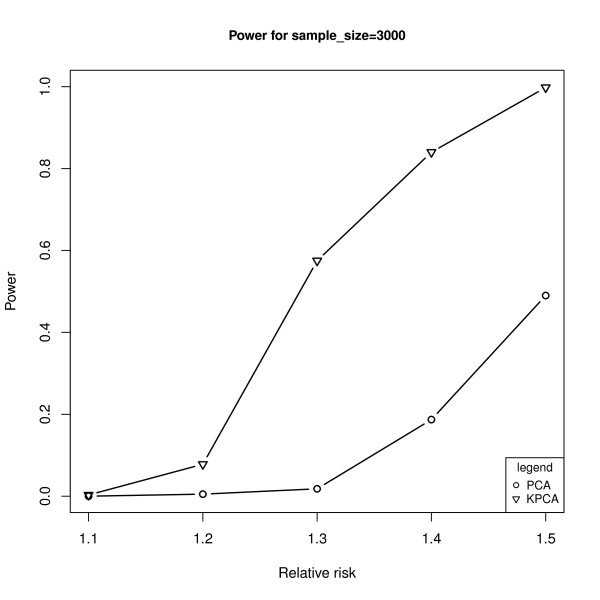
**The powers of PCA-LRT and KPCA-LRT under different relative risks at the given of sample sizes 3000**. The horizontal axis denotes the relative risks and the vertical axis denotes the powers of PCA-LRT and KPCA-LRT.

**Figure 5:**
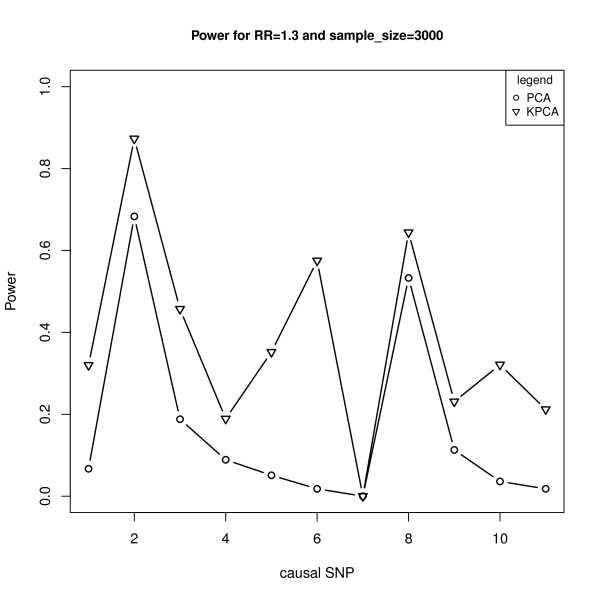
**The powers of PCA-LRT and KPCA-LRT at the given sample size of 3000 and relative risk of 1.3 when each of the 11 SNPs was set as the causal variant**. The horizontal axis denotes the positions of the causal variant and the vertical axis denotes the powers of PCA-LRT and KPCA-LRT.

With the same causal variant as above, Figure 3 shows the powers of the two models under different sample sizes at the given relative risk of 1.3, while Figure 4 shows the powers under different relative risks at the given sample size of 3000. As expected, the powers are monotonically increasing functions of sample sizes and the relative risk levels for both models. Furthermore, the powers of KPCA-LRT are much higher than PCA-LRT when the sample size is not less than 3000 (Figure 3). Both models are less powerful when RR is less than 1.2. At higher relative risks, KPCA-LRT also shows greater power than PCA-LRT. Especially at the relative risks of 1.3, the power of PCA-LRT is close to zero while it is about 0.6 for KPCA-LRT (Figure 4). Figure 5 shows the powers of both models at the given sample size of 3000 and relative risk of 1.3 when each of the 11 SNPs is set as the causal variant. Interestingly, KPCA-LRT is always more powerful than PCA-LRT in each case.

These simulation results indicate that the powers of KPCA-LRT are always higher than PCA-LRT at given significant levels, sample sizes and relative risks. Particularly, under lower relative risk (1.2 and 1.3) and smaller significant levels (1E-5 and 1E-7), KPCA-LRT is more powerful than PCA-LRT.

### Application

Table 2 shows the information of the selected four regions and the performances of PCA-LRT, KPCA-LRT and single-locus test. For region 1, the statistical significances at the given nominal level (1E-5) were detected by all the three methods. For region 2, the same significance was found by both single-locus test and KPCA-LRT, while PCA-LRT did not identify this region. Only the KPCA-LRT detected the significance for region 3, and both PCA-LRT and KPCA-LRT identified significance for region 4. These results suggested that KPCA-LRT performs the best among the three methods.

**Table 2:** The performances of single-locus test, PCA-LRT and KPCA-LRT

Region	# of SNPs	Physical location	Gene involved	Results		
				
				Single**	PCA	KPCA
Region 1	12	114030646-114132504	PTPN22	2.30E-8*	4.63E-9*	3.14E-9*
Region 2	8	143025126-143050638	ANKRD35	1.94E-6*	0.837	4.25E-6*
Region 3	13	156523590-156572131	DUSP23	2.47E-4	6.01E-3	7.82E-6*
Region 4	15	19880889-19929909	RNF186	2.05E-4	5.33E-6*	2.54E-6*

*significant at the level of 1E-5

**the most significant p value in the corresponding region

## Discussion

In genetic association study, especially in GWAS, in order to avoid the collinearity among SNPs and reduce the false positive rate caused by multiple testing, several groups have proposed PCA-based methods and found that these methods are typically as or more powerful than both single locus test and haplotype-based test [20-23]. However, it is not enough to just consider the linear relationship between SNPs, and the PCA-based methods will lose power when the nonlinear relationship exists in the genome. In this paper, based on the ideas of Wu et al [32] and Liu et al [32], we combined KPCA with LRT to propose the KPCA-LRT model for detecting the association between multiple SNPs and diseases. The simulation results (Table 1, Figure 2 to Figure 5) showed that KPCA-LRT performed well under null hypothesis, and all the powers of KPCA-LRT were higher than PCA-LRT at given significant levels, sample sizes and relative risks, especially under lower relative risk (1.2 and 1.3) with smaller significant levels (1E-5 and 1E-7). Specifically, we set five low levels of relative risks (1.1-1.5) because the great majority of the identified risk marker alleles conferred very small relative risks [46]. Our simulation results show that KPCA-LRT is much more powerful than PCA-LRT when the sample size is not less than 3000 (Figure 3). Both models are less powerful when RR is lower than 1.2. At higher relative risks, KPCA-LRT also shows greater power than PCA-LRT. Especially at the relative risks of 1.3, the power of PCA-LRT is close to zero while it is about 0.6 for KPCA-LRT (Figure 4). To investigate the performance of the two methods on different causal SNPs with different MAF and different LD patterns, each of the 11 SNPs is defined as the causal variant. In each case, KPCA-LRT is more powerful than PCA-LRT (Figure 5).

To compare the three methods (single-locus test, PCA-LRT and KPCA-LRT), the four regions from the RA data in GAW16 Problem 1 (Table 2) are considered in this paper. For region 1, the statistical significances at the given nominal level (1E-5) were detected by all three methods. For region 2, the same significance is found by both single-locus test and KPCA-LRT, while PCA-LRT did not identify this region. There are no reports on the association of region 3 and region 4, but in this paper the results of KPCA-LRT show that there may be susceptible locus in the two regions, and the result of PCA-LRT on region 4 coincided with KPCA-LRT. In conclusion, KPCA-LRT performed the best among the three methods.

The four genes involved in the regions for real data analysis are selected based on prior researches and Gene Ontology [47]. The definition of "region" is very broad, such as a single SNP, a haplotype, a gene set, or interval of constant copy number [8]. To be easily interpreted, genes or genome regions are often defined based on the biological knowledge, such as Gene Ontology and KEGG [48]. For large genes or regions, it is hard to fine map the causal SNPs or associated markers even if association between the whole genes or regions could be detected. Recently sliding-window scan approaches have been widely used to partition the large genes or regions into many overlapping/non-overlapping regions [49,50]. Then the proposed gene- or region-based methods can be used in each region.

There are several limitations about the proposed method. First, only one causal SNP is considered in present work. Second, how to fix the kernel function with appropriate parameters for each data is still a theoretical problem. Third, when the effect size is smaller (relative risk per allele = 1.1, see Figure 3), both PCA-LRT and KPCA-LRT are less powerful. Fourth, all the frequencies of the causal SNPs are higher than 0.05, so it is hard to decide whether the proposed method is powerful for rare variants. The last, the proposed KPCA-LRT is based on logistic regression, so it could not deal with quantitative traits. To do this, KPCA-based methods could be combined with e.g. multivariate regression analysis or partial least squares (PLS) [51]. Further work to solve such problems will certainly be warranted.

## Conclusions

In present study, we have proposed a KPCA-LRT model for testing associations between a candidate gene or genome region with diseases (or traits). Results from both simulation studies and application to real data show that KPCA-LRT with appropriate parameters is always as or more powerful than PCA-LRT, especially under lower relative risks and significant levels.

## Competing interests

The authors declare that they have no competing interests.

## Authors' contributions

QSG, YGH, ZSY, JHZ, BBZ and FZX conceptualized the study, acquired and analyzed the data and prepared for the manuscript. All authors approved the final manuscript.
